# Supplementary material for: MOF-in-COF molecular sieving membrane for selective hydrogen separation
Source: Nat Commun. 2021 Jan 4;12:38. doi: 10.1038/s41467-020-20298-7 (PMC7782778; doi:10.1038/s41467-020-20298-7)
Supplement: Supplementary file 1 — Supplementary Information [file 41467_2020_20298_MOESM1_ESM.pdf]

Supplementary Information

MOF-in-COF molecular sieving membrane for selective hydrogen separation

Fan et al.

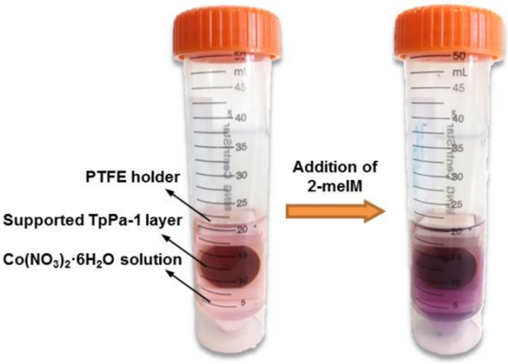

Supplementary Figure 1. Optical photograph of two-stage immersion process.

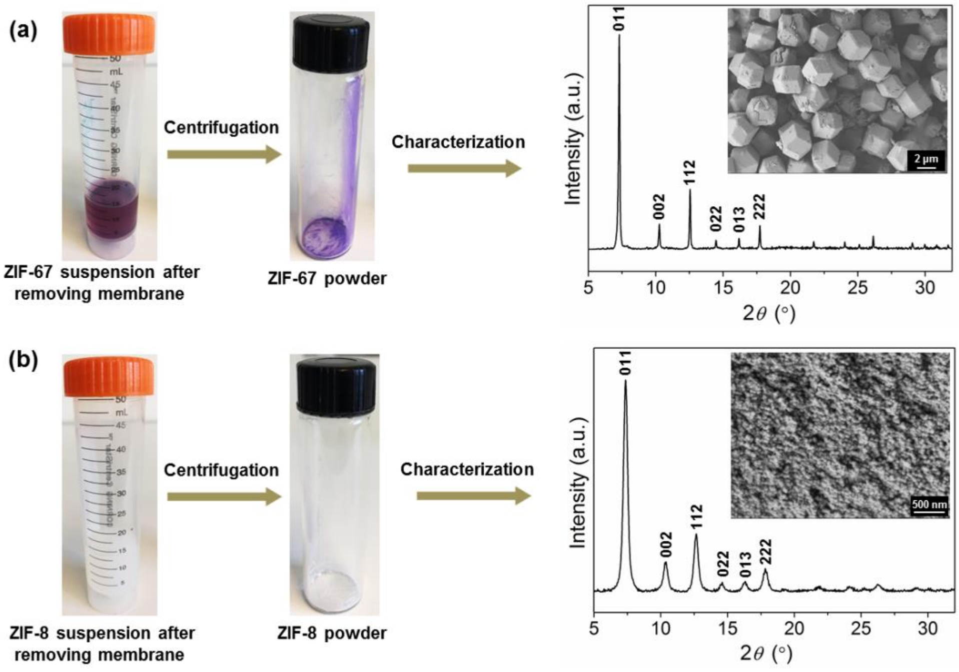

Supplementary Figure 2. XRD and SEM characterization of (a) ZIF-67 and (b) ZIF-8 crystals in suspension after synthesis of MOF-in-COF membranes.

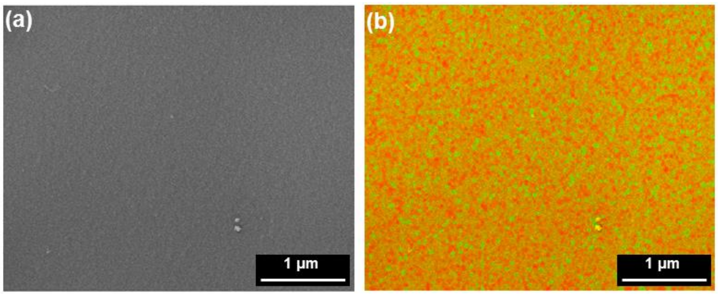

Supplementary Figure 3. (a) Surface SEM image and (b) corresponding EDXS mapping of the ZIF-67-in-TpPa-1 membrane. C Kα1_2, red; Co Kα1, green.

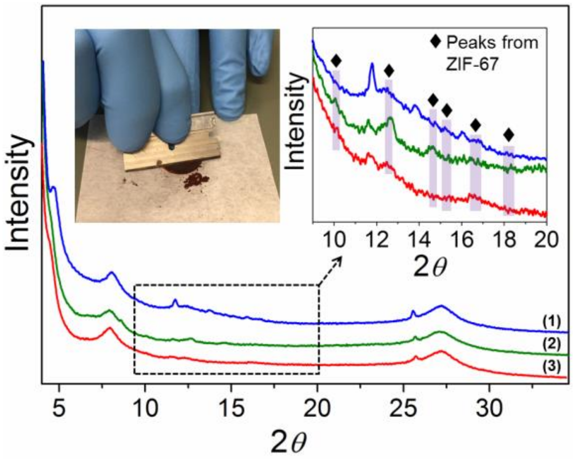

Supplementary Figure 4. XRD patterns of MOF-in-COF powders scraped from the substrate with the insets of scraping photograph (left) and magnified zone (right). To ensure the measurement accuracy, the MOF-in-COF powers were collected from at least 10 parallel membrane samples, and then divided into three (1, 2, 3) parts for XRD.

Different from the previous XRD characterization of supported MOF-in-COF membrane shown in Fig. 2a, the XRD patterns of the powdered MOF-in-COF samples scraped from the α-Al_2O_3 substrate were measured (see Supplementary Fig. 4). By measuring the XRD of the scraped off powder, the strong XRD signals of the alumina support can be avoided. To intensify the XRD diffraction peaks of ZIF-67 as much as possible, samples of sufficient mass were needed. Therefore, we collected powders from at least 10 MOF-in-COF membranes in parallel. To ensure the accuracy, the scraped MOF-in-COF powders were divided into three groups for the XRD measurement. By optimizing the test parameters, it is expected that some XRD reflections related to ZIF-67 could be captured. The measurement result is shown in Supplementary Fig. 4. It can be seen that there are several diffraction peaks assigned to the ZIF-67 appearing in the magnified XRD zone, despite they are still not very strong. This clearly indicates the formation of ZIF-67 most probably inside the COF layer. Of course, considering the weak XRD diffractions, we can not exclude there have been amorphous ZIF-67 in the confined COF layer. Even so, the formed microporous network in the nano-sized COF channel is still transport-active and improves the hydrogen separation selectivity compared to the individual COF membrane.

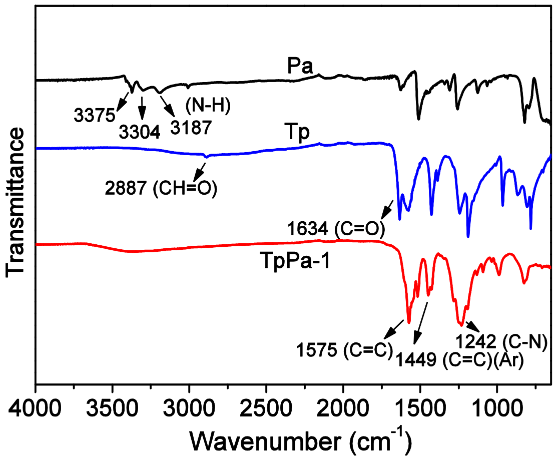

Supplementary Figure 5. FT-IR spectra of the Pa, Tp and the as-synthesized TpPa-1 powder collected in the same autoclaves as the membrane.

The spectra for TpPa-1 show the almost disappearance of the characteristic N−H stretching band of the free diamine (3100−3400 cm^{-1} from Pa) and of the aldehyde (1634 cm^{-1} from Tp), indicating the complete consumption of monomers. The peaks at 1449 cm^{-1} and 1242 cm^{-1}, are assigned to the aromatic C=C (Ar) and C-N bond in the keto-enamine form of TpPa-1. Simultaneously, the absence of hydroxyl (O−H) and C=N stretching peaks and the appearance of new C=C peak at 1575 cm^{-1} reveal the keto-enamine form of TpPa-1.

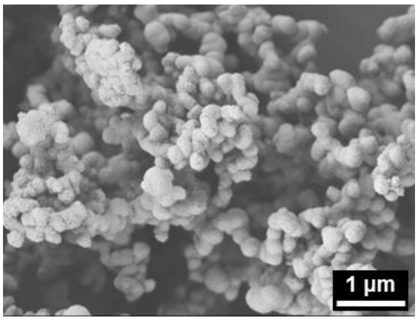

Supplementary Figure 6. SEM image of the as-synthesized TpPa-1 powder collected in the same autoclaves as the membrane.

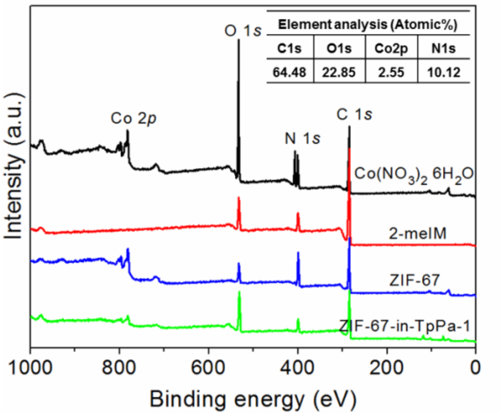

Supplementary Figure 7. Typical XPS survey spectra with inserted elemental analysis.

The MOF volume fraction (vol%) could be obtained based on the Co/O atomic (molar) ratio measured by XPS on the MOF-in-COF selective layer. According to the chemical formula of ZIF-67 (Co (2-MeIM)$_2$)) and TpPa-1 ($C_{18}H_{12}N_3O_3$), and their densities (0.903 and ~ 0.6 g/cm^3, respectively), the estimated loading of ZIF-67 in the MOF-in-COF matrix is about 13.3 vol%.

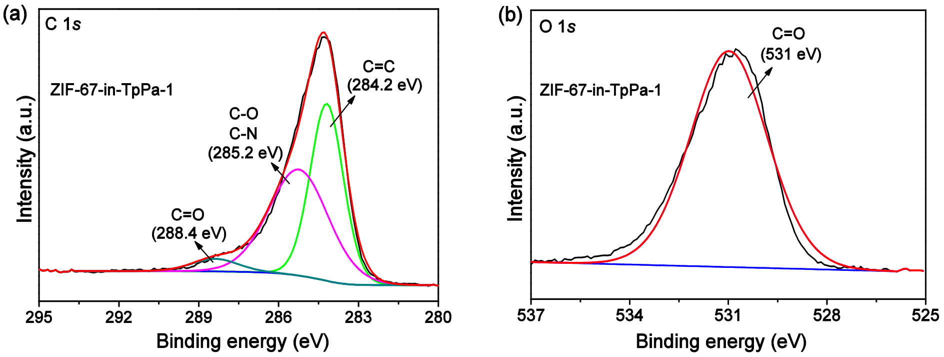

Supplementary Figure 8. High-resolution XPS spectra of deconvoluted C1*s* (a) and O1*s* (b) in the ZIF-67-in-TpPa-1 membrane.

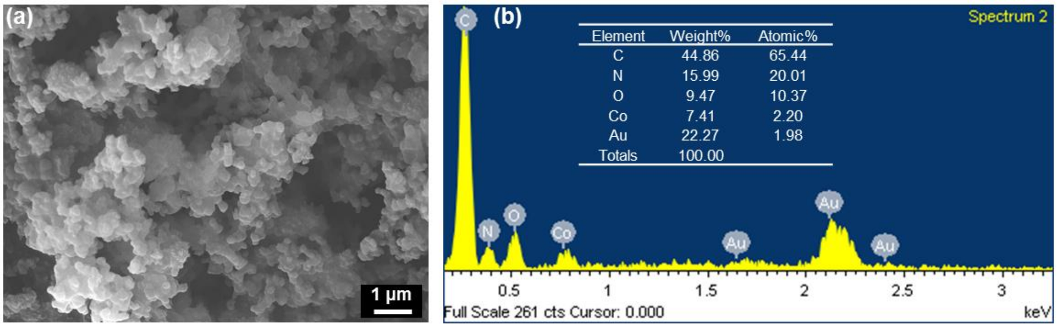

Supplementary Figure 9. (a) SEM image and (b) corresponding EDXS elemental analysis of ZIF-67-in-TpPa-1 suspension.

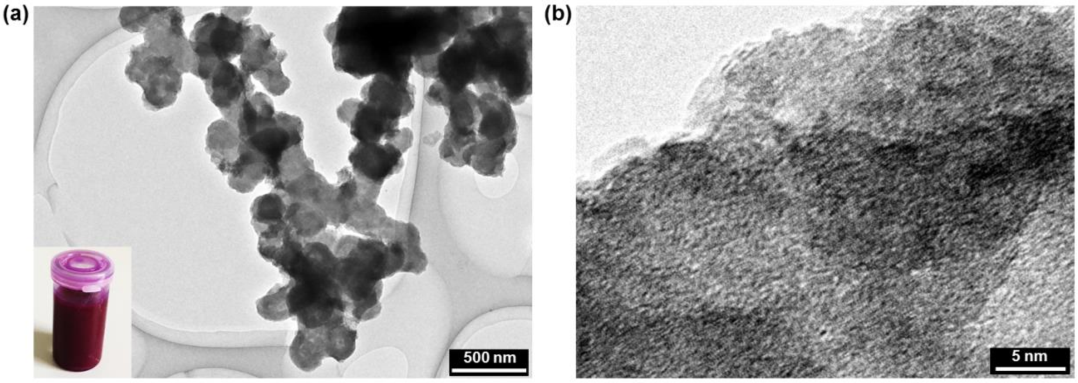

Supplementary Figure 10. (a) TEM image with inserted digital photograph of ZIF-67-in-TpPa-1 suspension (left-bottom corner). (b) High-resolution TEM image of the ZIF-67-in-TpPa-1 in suspension.

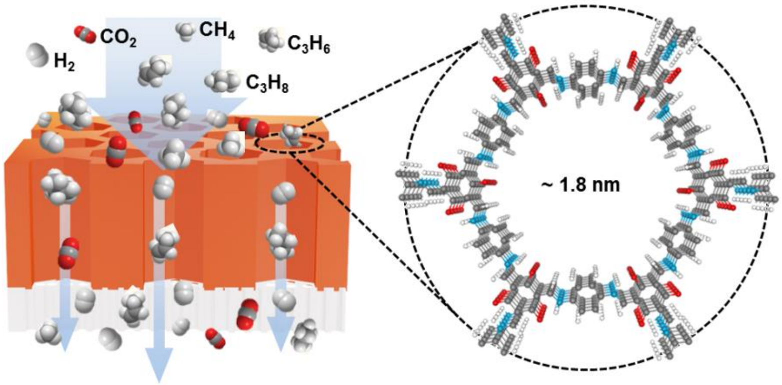

Supplementary Figure 11. Schematic illustration of the gas transport through non-modified TpPa-1 membrane.

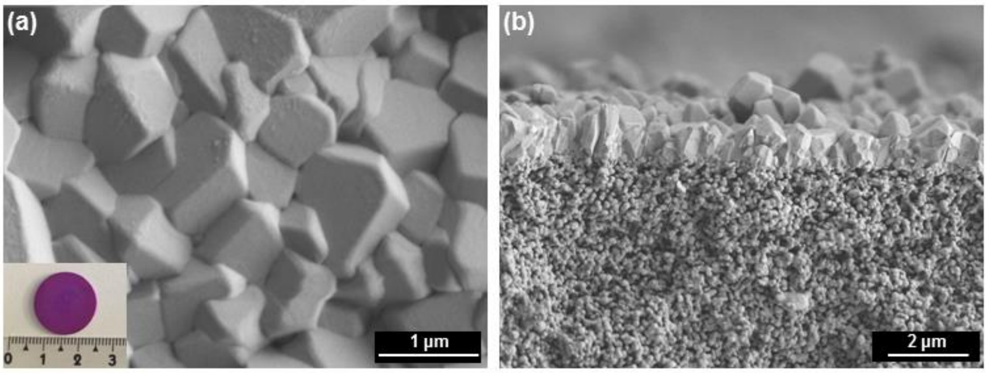

Supplementary Figure 12. (a) Surface and (b) cross-sectional SEM images of as-synthesized ZIF-67 membrane. Insert shows the optical photograph of membrane.

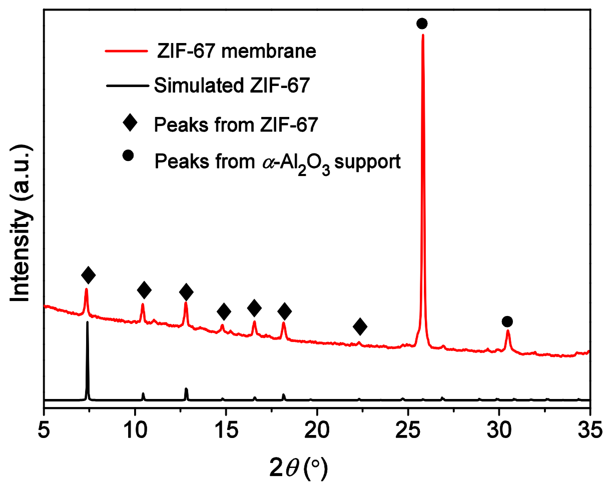

Supplementary Figure 13. XRD patterns of as-synthesized ZIF-67 membrane and simulated ZIF-67.

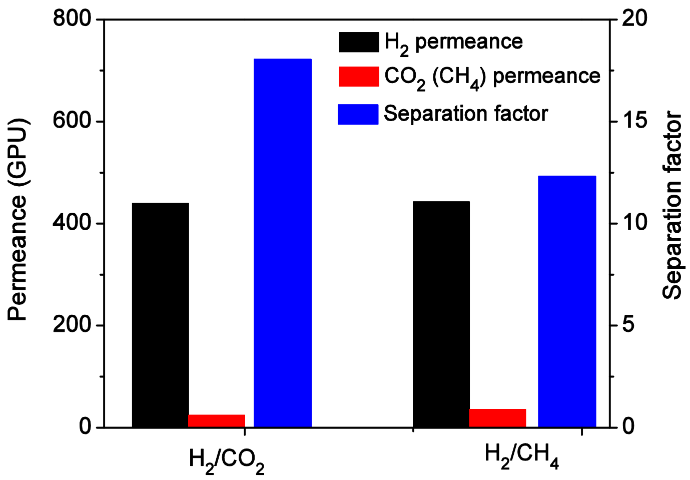

Supplementary Figure 14. Permeance and separation factors of the ZIF-67 membrane for equimolar binary gases at 298 K and 1 bar.

As shown in Supplementary Fig. 14, the ZIF-67 membrane has separation factors of about 18, and 13 for H_2/CO_2, and H_2/CH_4 gas pairs, respectively. Simultaneously, the H_2 permeance is about 440 GPU. Both selectivity and H_2 permeance are far below the MOF-in-COF membrane. The relatively low separation selectivities are as expected because there are void/low-density or nonselective regions easily existed in the MOF layer during the intergrowth process by the seeded-assisted in-situ solvothermal approach. Moreover, the gate opening flexibility in ZIF-67 would also result in the poor molecular sieve performance. The low permeance is mainly due to the intrinsic ultramicropore system of ZIF-67 and perhaps as a sequence of the formation of impermeable regions caused by the intergrowth of crystals.

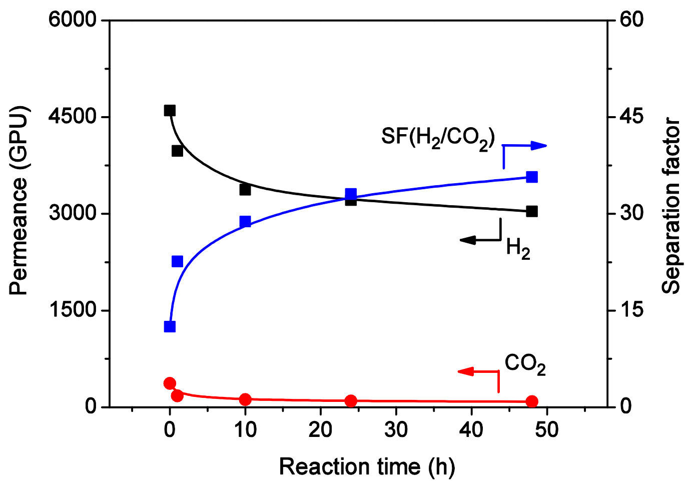

Supplementary Figure 15. Changes of gas permeance performance with the reaction time for ZIF-67 after addition of 2-meIM. (Equimolar binary gases at 298 K and 1 bar.)

As shown in Supplementary Fig. 15, the separation selectivity of the H_2/CO_2 mixture increased dramatically within 10 h after addition of 2-meIM, and after 24 h there was only a slight further increase. The H_2 permeance decreased gradually and then leveled off. For example, the H_2/CO_2 selectivity was only 12.5 for the pristine Co^{2+}@TpPa-1 membrane (without addition of 2-meIM). It has a more than two-fold improvement (28.2) for the MOF-in-COF membrane within 10 h, but subsequently, the selectivity increased not too much, only from 33 to 35 by extending the synthesis time from 24 h to 48 h. This suggests the MOF-in-COF pore structure has been formed in each 1D channel of COFs after 24 h.

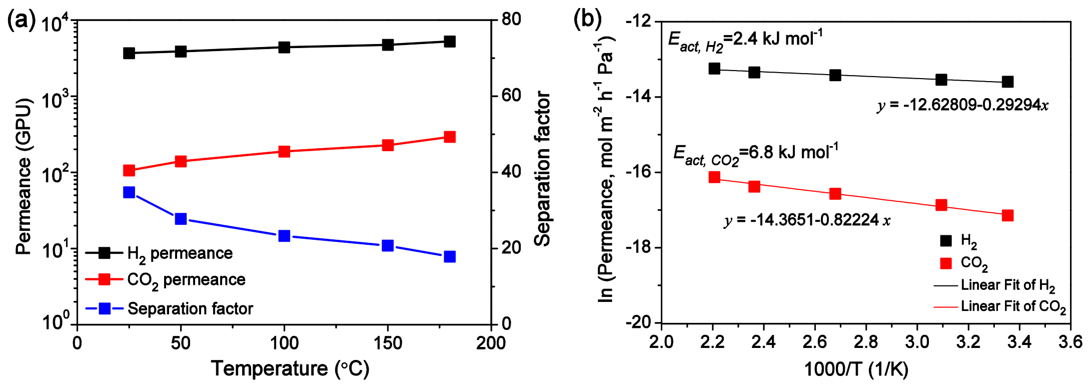

Supplementary Figure 16. (a) H_2/CO_2 separation factor and H_2 permeances of the ZIF-67-in-TpPa-1 membrane as function of the temperature at 1 bar. (b) Arrhenius temperature dependence of H_2 and CO_2 permeance through the ZIF-67-in-TpPa-1 membrane.

As shown in Supplementary Fig. 16a, with the increase in test temperature from room temperature to

180 ℃, the H_2/CO_2 separation factor gradually decreases from 34.8 to 18. This can be explained from the apparent activation energy (E_{act}) for the H_2 and CO_2 which are calculated from an Arrhenius equation, as follows (eq. 1).

$$P_i = A_0 \exp(-\frac{E_{act,i}}{RT}) \qquad (1)$$

where P_i, A_0 and $E_{act,i}$ represent the gas permeance, pre-exponential factor and the activation energy of component i, respectively. R and T are the ideal gas constant and absolute temperature (K), respectively. The ln (P_i) versus 1/T displays a linear correlation, and $E_{act,i}$ can be determined from the slope. As shown in Supplementary Fig. 16b, the calculated $E_{act,CO2}$ value is 6.8 kJ mol^{-1} higher than that of H_2 (2.4 kJ mol^{-1}).

The apparent activation energy ($E_{act,i}$) is related to the diffusion activation energy (E_{diff}) and adsorption heat (Q_{st}).

$$E_{act,i} = E_{diff} - Q_{st} \qquad (2)$$

The adsorption heat of CO_2 is expected to exhibit higher value than that of the H_2, considering the stronger adsorption of CO_2 in COFs and ZIFs. Therefore, the diffusion activation energy of CO_2 is also expected to be higher than H_2. This indicates much more activated diffusion of CO_2 through MOF-in-COF membrane at a higher permeation temperature, which leads to the decrease of H_2/CO_2 separation factor.

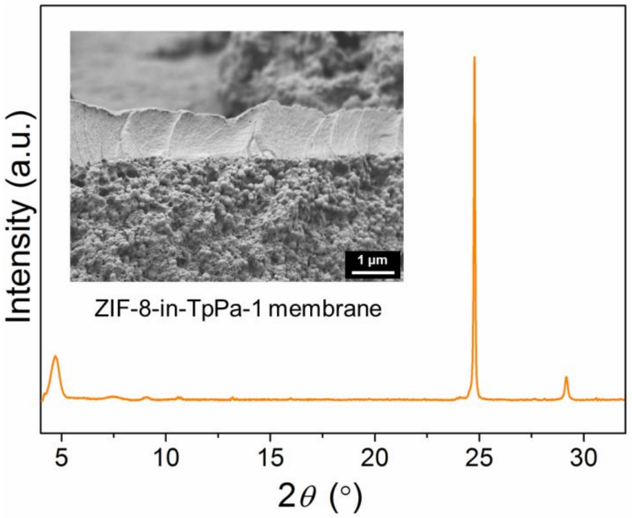

Supplementary Figure 17. XRD pattern with inserted cross-sectional SEM image of ZIF-8-in-TpPa-1 membrane.

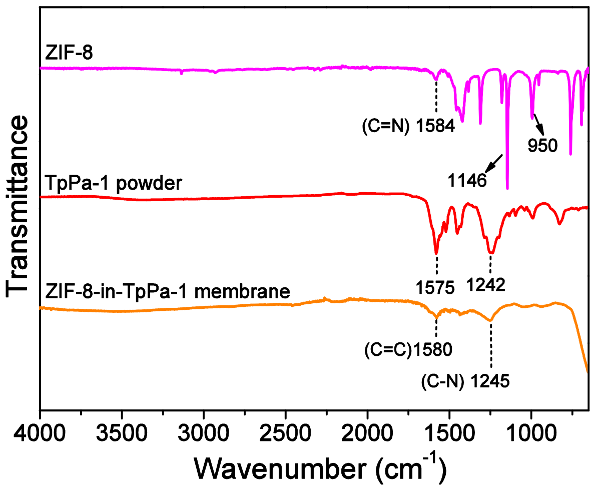

Supplementary Figure 18. FT-IR spectra of as-synthesized ZIF-8 powder, TpPa-1 powder and ZIF-8-in-TpPa-1 membrane.

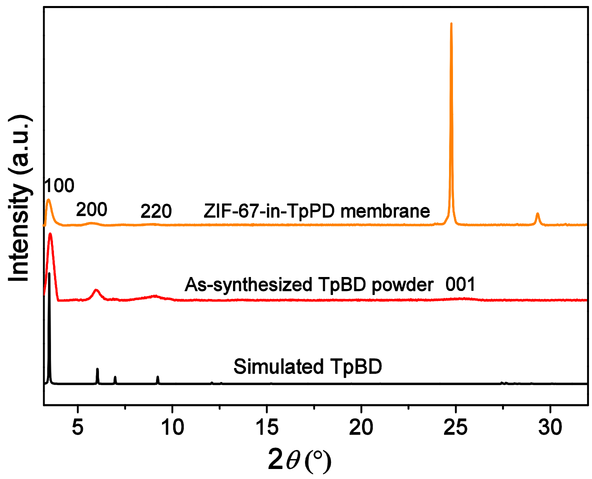

Supplementary Figure 19. XRD patterns of ZIF-67-in-TpBD membrane and TpBD powder collected in the same autoclaves as the membrane.

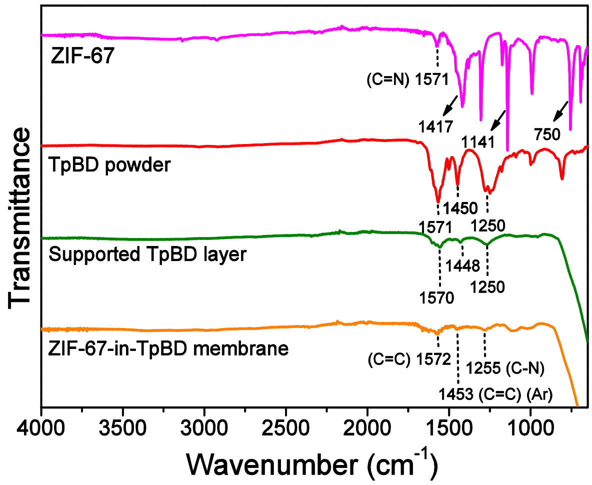

Supplementary Figure 20. FT-IR spectra of as-synthesized ZIF-67 powder, TpBD powder, TpBD layer and ZIF-67–TpBD membrane.

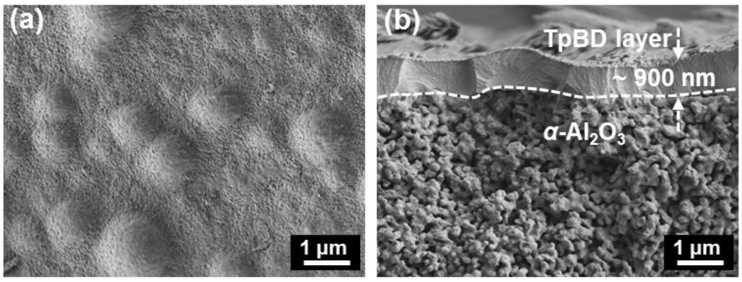

Supplementary Figure 21. (a) Top-view and (b) cross-sectional SEM image of the prepared α-Al_2O_3 supported TpBD layer.

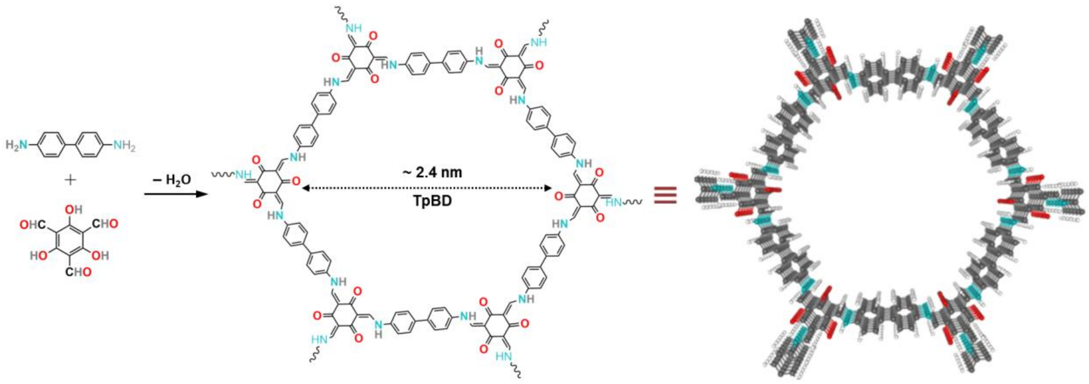

Supplementary Figure 22. Schematic of synthesis and chemical structure of TpBD

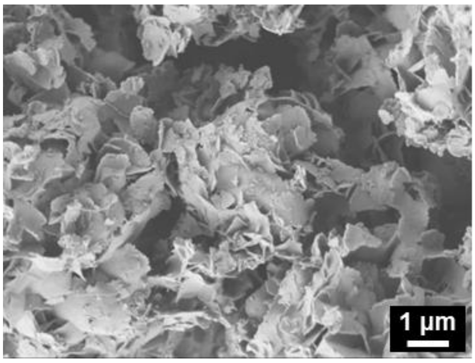

Supplementary Figure 23. SEM image of TpBD powder collected in the same autoclaves as the membrane.

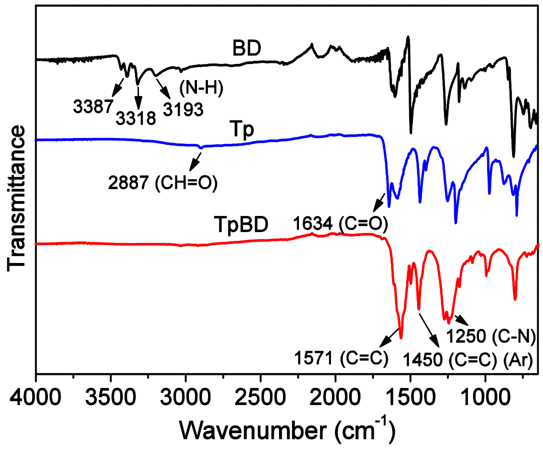

Supplementary Figure 24. FT-IR spectra of the BD, Tp and the synthesized TpBD.

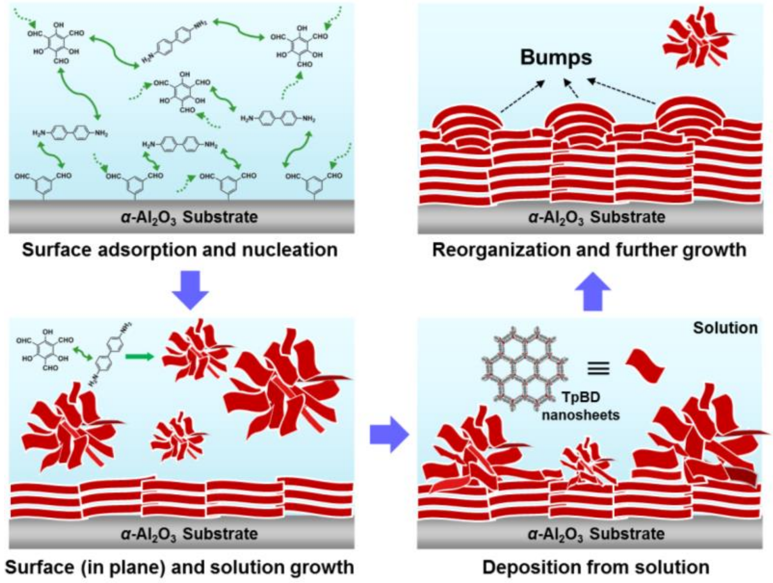

Supplementary Figure 25. Schematic illustration of formation process of TpBD bumps on the surface..

SEM images show the TpBD bumps on the surface of the membrane (see Fig. 4f and Supplementary Fig. 21). The forming process is illustrated in the schematic of Supplementary Fig. 25**.** Initially, the reactant molecules of 1,3,5-triformylbenzene (Tp) and benzidine (BD) in solution are adsorbed to the aldehyde-modified α-Al_2O_3 surface. Due to the lower interfacial energy and thermodynamic barrier, it would lead to kinetically faster surface nucleation and promote in plane lateral growth of TpBD[1]. In this process, in the solution the non-adsorbed monomers are also nucleated and grown into the multilayers either through stacking of metastable nanosheets, or through template growth. Once crystallites are formed, these 2D multilayers are easily aggregated into the polycrystallites in shape of near-spherical structure under the effect of surface tension and interaction between multilayers[2-4]. Subsequently, these

solution-grown polycrystallites consisting of 2D TpBD nanosheets adhere or deposit on top of previous COF layers. Furthermore, through the error-checking and self-correction from the templating effect of the underlying surface layers, they are allowed to rearrange and grow as new layers along the surface normal. But the spherical polycrystallites which have not enough time to completely reinstate the in-plane orientation to the substrate will form the bumps on the surface.

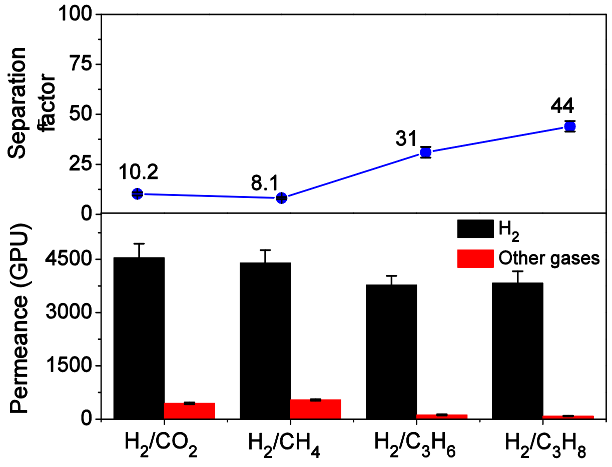

Supplementary Figure 26. Permeance and separation factors of the non-modified TpBD membrane for equimolar binary gases at 298 K and 1 bar.

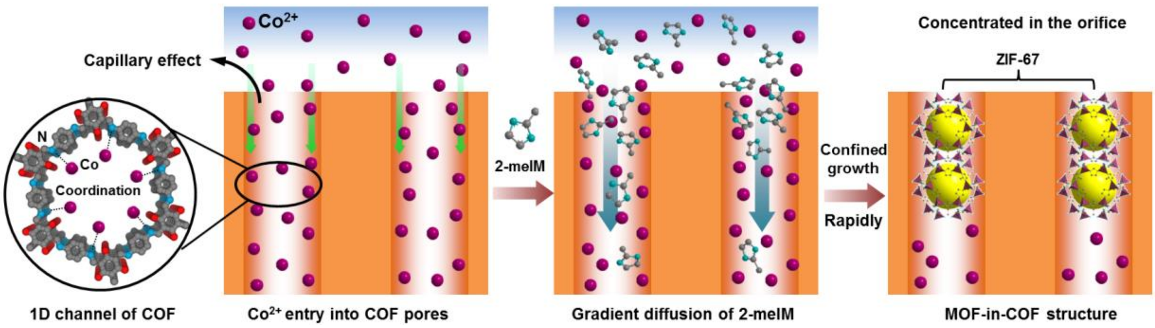

Supplementary Figure 27. Schematic illustrating the formation process of MOF-in-COF structure.

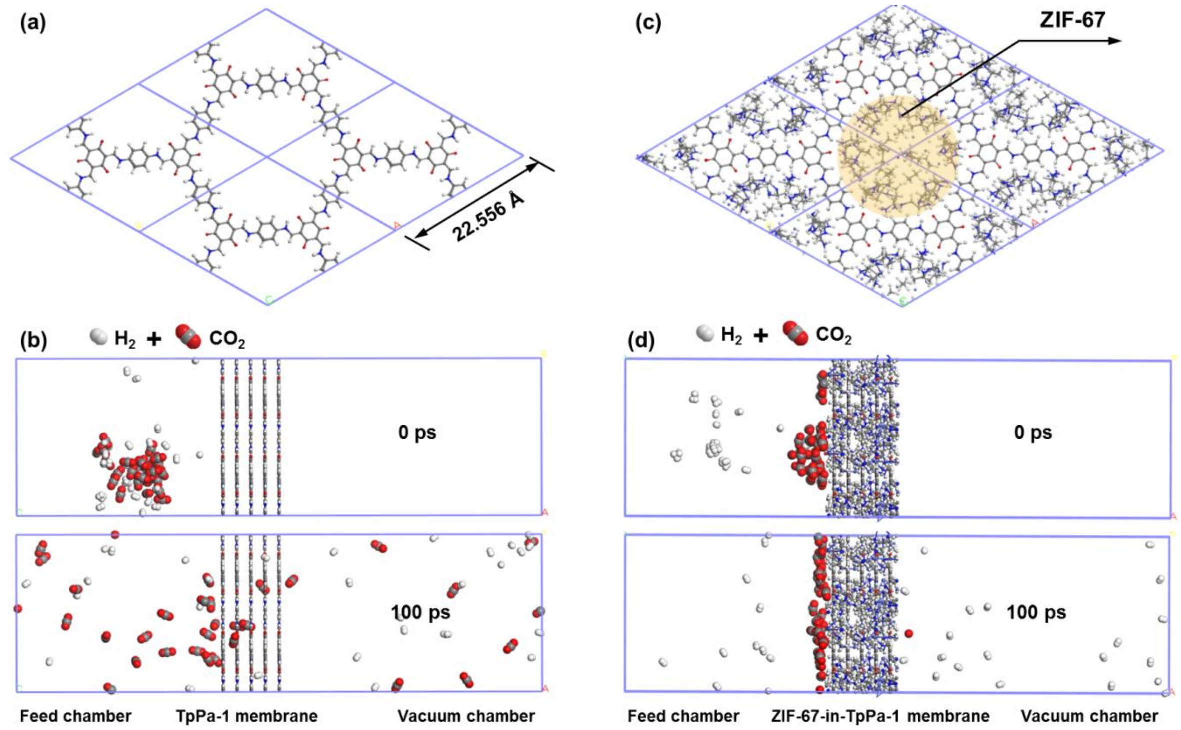

Supplementary Figure 28. Atomic structure of (a) TpPa-1 and (b) ZIF-67-in-TpPa-1. Simulation system with snapshot at 0, 100 ps for the permeation of an equimolar H_2/CO_2 (30 H_2 and 30 CO_2 molecules in the feed chamber) mixture through (a) TpPa-1 membrane and (b) ZIF-67-in-TpPa-1 membrane.

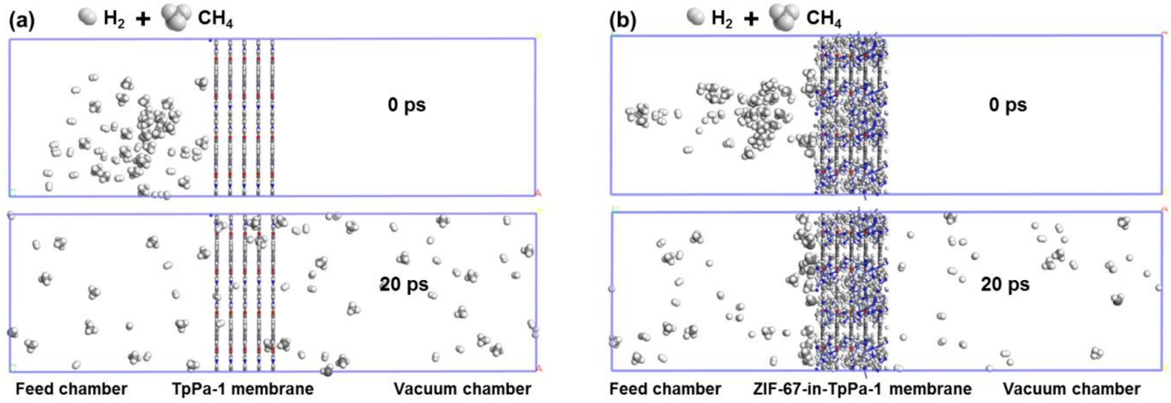

Supplementary Figure 29. Molecular structure of (a) TpPa-1 and (b) ZIF-67-in-TpPa-1. Simulation system with snapshot at 0, 100 ps for the permeation of an equimolar H_2/CO_2 (30 H_2 and 30 CH_4 molecules in the feed chamber) mixture through (a) TpPa-1 membrane and (b) ZIF-67-in-TpPa-1 membrane.

3. Supplementary Tables (Table 1 to Table 3)

Supplementary Table 1 Single and equimolar mixture gas permeances (GPU) and separation factors for the ZIF-67-in-TpPa-1 membrane at 298 K and 1 bar.

$Gas_{i/j}$	Knudsen constant	Performance of the ZIF-67-in-TpPa-1 membrane					
		Single gas			Mixed gases		
		Permeance (i) (GPU)	Permeance (j) (GPU)	Ideal separation factor	Permeance (i) (GPU)	Permeance (j) (GPU)	Separation factor
H_2/CO_2	4.7	3752.1	97.8	38.3	3671.1	105.3	34.9
H_2/CH_4	2.8		99.2	37.8	3252.7	97.7	33.3
H_2/C_3H_6	4.6		31.8	117.7	3347.2	30.3	110.5
H_2/C_3H_8	4.7		18.1	206.8	3212.2	16.6	192.7

Supplementary Table 2 A summary of the H_2/CO_2 separation performance of various membranes.

Membrane materials		Thickness (μm)	Temperature (℃)	H_2 permeance (GPU)	Separation factor (H_2/CO_2)	Reference
Zeolite	$AlPO_4$	6	35	990.3	9.7	5
	A-type	3.8	35	294.7	9.4	6
	DDR+CVD	15	550	58.9	32.7	7
	LTA	10	20	2387.4	10.9	8
	LTA (ITQ-29)	12	300	1178	7.8	9
	LTA (triple layer)	10.5	100	471.6	12.5	10
	LTA ($ALPO_4$)	25	200	560	10.9	11
	MFI+CVD	2	450	442.1	8.2	12
	MFI+CCD	2	500	29.5	45.6	13
	NaA	5	65	1491.4	92	14
	SAPO-34	26	30	26526.8	16.7	15
	SAPO-34/Pd (CVD)	25	30	4362.2	20.8	16
	B-ZSM-5+MDES	3.5	400	294.7	47	17
	ZSM-5/Silicalite-1	10	500	353.7	23	18
	ZSM-5/Silicalite-1	9.5	25	377.3	25.3	19
MOF	HKUST-1	60	25	2947.4	6.8	20
	ZIF-7	1.5	200	227.2	6.48	21
	ZIF-22	40	50	489.3	7.2	22
	NH_2-MIL-53 (Al)	15	15	5850.6	30.9	23
	Amine-Mg-MOF-74	10	25	224	28	24
	ZIF-7	2	200	27.1	8.4	25
	ZIF-8	12	25	294.7	6	26
	ZIF-8	2	25	1267.4	3.28	27
	MIL-96 (Al)	8	RT	1532.6	8.8	28
	$Zn_2(Bim)_3$	0.01	200	1918.8	128.4	29
POM	PIM-EA-TB	181	25	42.1	1.09	30
	PIM-SBI-TB	157	25	138.2	0.76	30
	mPBO	--	210	5.1	6.2	31
	PHBOA (8:2)	--	210	1	8	32
	Polybenzimidazole (PBI)	--	270	3.2	20	33,34
	Pure PBI	--	35	1.8	8.6	35
	Pure PBI	--	35	0.058	7.1	36
	PBI	10	150	2.6	16	37
	BILP-101x (400 nm)	0.4	150	24.2	39.5	38
	NUS-2@PBI	50-100	35	0.059	18.78	39

	[COF-300]-[Zn2(bdc)2(dabco)]	97	RT	1344	12.6	40
	[COF-300]-[ZIF-8]	100	RT	1055.2	13.5	40
	COF-300	40	RT	2688	6.0	40
	COF-LZU1	0.5	25	3684.3	6.0	41
	ACOF-1	0.6	25	2019	14.1	41
	COF-LZU1–ACOF-1	1	25	660.2	24.2	41
	CTF-1	0.1	RT	5010.6	17.4	42
	TpPa-1-30/GO-10	0.3	25	3144.9	25.57	43
	[COF-300]-[UiO-66]	100	RT	1173.1	17.2	44
ZIF-67-in-TpPa-1		1	25	3671.1	34.9	This work
ZIF-8-in-TpPa-1		1	25	3920.1	23.1	
ZIF-67-in-TpBD		0.9	25	3772.7	27.9	

POM: Porous organic materials; MOFs: Metal-organic frameworks; PIMs: Polymers of intrinsic microporosity; TR polymer: Thermally rearranged polymer; PBI: Polybenzimidazole; POFs: Porous organic frameworks.

Supplementary Table 3 A summary of the H_2/CH_4 separation performance of various membranes.

Membrane materials		Thickness (μm)	Temperature (℃)	H_2 permance (GPU)	Separation factor (H_2/CH_4)	Reference
Zeolite	AIPO-18	4-10	25	265.3	18.7	45
	FAU	2.7	50	551.2	9.9	46
	FAU	2.5	100	1179	4	47
	Ag-LTA	4.5	50	677.9	21.3	48
	SAPO-34	15	25	70.7	25	49
	SAPO-34	10	200	159.2	7.9	50
	SAPO-34	4	25	20514	14.7	51
	SAPO-34/Carbon	2	25	294.7	97	52
	SSZ-13	4-6	200	533.4	16	53
	SSZ-13+CLD	5-6	25	1040.4	5.9	54
	Zeolite L/Carbon	3-4	25	294.7	62.6	55
	Zeolite L/Carbon	3-4	25	26.5	103	56
MOF	HKUST-1	3	RT	5924.3	5.4	57
	HKUST-1	20	RT	884.2	8.8	58
	ZIF-7-8	2	25	250.5	11.4	59
	ZIF-8	0.5	25	24463.6	16.2	60
	ZIF-8	1	RT	59243.1	9.1	61
	ZIF-8	2	RT	1797.9	10.2	62
	ZIF-9	30	25	1606.3	10.35	63
	ZIF-90	20	200	571.8	18.9	64
	ZIF-90	20	225	854.7	70.5	65
	ZIF-94	7	35	47.2	85.6	66
	ZIF-8	35	35	589.5	10.5	67
	ZIF-8	30	25	178	11.2	68
	ZIF-8	20	150	648.4	31.5	69
	ZIF-8	2	RT	4539	13	70
	ZIF-8	6	RT	468.6	11.4	71
	ZIF-8	20	RT	433.3	12.5	72
	ZIF-90	20	225	840	70.5	73
	2abIm-VPLT-RTD-ZIF-8	3	25	442.1	140	74
POM	PIM-EA-TB	181	25	42.1	11.1	30
	PIM-SBI-TB	157	25	138.2	4.9	30
	TPIM−1	--	25	87	53	75
	TPIM−2	--	25	21.5	36	75

	CoPI-TB-4	--	35	21.9	37	76
	CC3	0.05	RT	4509.5	16.5	77
	TpPa-1(40)@PBI-BuI	56.5	35	0.327	165.5	78
	TpBD(50)@PBI-BuI	70.24	35	0.595	139.7	78
	COF-320	4	RT	1671.2	2.5	79
	COF-LZU1	0.5	25	3389.5	9.65	41
	ACOF-1	0.6	25	1744.8	24.67	41
	COF-LZU1–ACOF-1	1	25	536.4	100.2	41
ZIF-67-in-TpPa-1		1	25	3252.7	33.3	This work
ZIF-8-in-TpPa-1		1	25	3743.2	22.1	
ZIF-67-in-TpBD		0.9	25	3566.4	24.9	

Supplementary References

1. Wang, H., He, B., Liu, F., Stevens, C., Brady, M. A., Cai, S., Wang, C., Russell, T. P., Tan, T. W. & Liu, Y. Orientation transitions during the growth of imine covalent organic framework thin films. *J. Mater. Chem. C.* **5**, 5090 (2017).

2. Wang, S., Zhang, Z., Zhang, H., Rajan, A. G., Xu, N., Yang, Y., Zeng, Y., Liu, P., Zhang, X., Mao, Q., He, Y., Zhao, J., Li, B. G., Strano, M. S. & Wang, W. J. Reversible Polycondensation-Termination Growth of Covalent-Organic-Framework Spheres, Fibers, and Films. *Matter* **1**, 1592–1605 (2019).

3. Koo, B. T., Heden, R. F. & Clancy. P. Nucleation and growth of 2D covalent organic frameworks: polymerization and crystallization of COF monomers. *Phys. Chem. Chem. Phys.* **19**, 9745–9754 (2017).

4. Smith, B. J. & Dichtel, W. R. Mechanistic Studies of Two-Dimensional Covalent Organic Frameworks Rapidly Polymerized from Initially Homogenous Conditions. *J. Am. Chem. Soc.* **136**, 8783–8789 (2014).

5. Guan, G., Tanaka, T., Kusakabe, K., Sotowa, K. I. & Morooka, S. Characterization of $AlPO_4$-type molecular sieving membranes formed on a porous α-alumina tube. *J. Membr. Sci.* **214**, 191–198 (2003).

6. Aoki, K., Kusakabe, K. & Morooka, S. Gas permeation properties of A-type zeolite membrane formed on porous substrate by hydrothermal synthesis. *J. Membr. Sci.* **141,** 197–205 (1998).

7. Zheng, Z., Hall, A. S. & Guliants, V. V. Synthesis, characterization and modification of DDR membranes grown on α-alumina supports. *J. Mater. Sci.* **43,** 2499–2502 (2008).

8. Li, X. et al. Ionothermal synthesis of LTA-type aluminophosphate molecular sieve membranes with gas separation performance. *Micropor.Mesopor. Mater.* **228,** 45–53 (2016).

9. Huang, A. & Caro, J. Steam-stable hydrophobic ITQ-29 molecular sieve membrane with H_2 selectivity prepared by secondary growth using Kryptofix 222 as SDA. *Chem. Commun.* **46,** 7748–7750 (2010).

10. Huang, A. Wang, N. & Caro, J. Synthesis of multi-layer zeolite LTA membranes with enhanced gas separation performance by using 3-aminopropyltriethoxysilane as interlayer. *Micropor.Mesopor. Mater.* **164,** 294–301 (2012).

11. Huang, A. & Caro, J. Highly oriented, neutral and cation-free AlPO4 LTA: from a seed crystal monolayer to a molecular sieve membrane. *Chem. Commun.* **47,** 4201–4203 (2011).

12. Zhu, X. Wang, H. & Lin, Y.S. Effect of the Membrane Quality on Gas Permeation and Chemical Vapor Deposition Modification of MFI-Type Zeolite Membranes. *Ind. Eng. Chem. Res.* **49,** 10026–10033 (2010).

13. Hong, Z. et al. Improvement of hydrogen-separating performance by on-stream catalytic cracking of silane over hollow fiber MFI zeolite membrane. *Int. J. Hydrogen Energy.* **38,** 8409–8414 (2013).

14. Dey, K. P., Kundu, D., Chatterjee, M. & Naskar, M. K. Preparation of NaA Zeolite Membranes Using Poly(Ethyleneimine) as Buffer Layer, and Study of Their Permeation Behavior. *J. Am. Ceram. Soc.* **96,** 68–72 (2013).

15. Das, J.K., Das, N. & Bandyopadhyay, S. Highly oriented improved SAPO 34 membrane on low cost support for hydrogen gas separation. *J. Mater. Chem. A* **1,** 4966–4973 (2013).

16. Das, J. K. & Das, N. Mercaptoundecanoic acid capped palladium nanoparticles in a SAPO-34 membrane: a solution for enhancement of H_2/CO_2 separation efficiency. *ACS Appl. Mater. Interfaces*. **6,** 20717–20728 (2014).

17. Hong, M., Falconer, J. L. & Noble, R. D. Modification of Zeolite Membranes for H_2 Separation by Catalytic Cracking of Methyldiethoxysilane. *Ind. Eng. Chem. Res.* **44,** 4035–4041 (2005).

18. Wang, H., Dong, X. & Lin, Y. Highly stable bilayer MFI zeolite membranes for high temperature hydrogen separation. *J. Membr. Sci.* **450,** 425–432 (2014).

19. Wang, H. & Lin, Y. Synthesis and modification of ZSM-5/silicalite bilayer membrane with improved hydrogen separation performance. *J. Membr. Sci.* **396,** 128–137 (2012).

20. Guo, H., Zhu, G., Hewitt, I. J. & Qiu, S. “Twin Copper Source” Growth of Metal−Organic Framework Membrane: $Cu_3(BTC)_2$ with High Permeability and Selectivity for Recycling H_2. *J. Am. Chem. Soc.* **131,** 1646–1647 (2009).

21. Li, Y. S. et al. Molecular sieve membrane: supported metal-organic framework with high hydrogen selectivity. *Angew. Chem. Int. Edit.* **122,** 548–551 (2010).

22. Huang, A., Bux, H., Steinbach, F. & Caro, J. Molecular-Sieve Membrane with Hydrogen Permselectivity: ZIF-22 in LTA Topology Prepared with 3-Aminopropyltriethoxysilane as Covalent Linker. *Angew. Chem. Int. Edit.* **49,** 4958–4961 (2010).

23. Zhang, F. et al. Hydrogen Selective NH_2-MIL-53(Al) MOF Membranes with High Permeability. *Adv. Funct. Mater.* **22,** 3583–3590 (2012).

24. Wang, N., Mundstock, A., Liu, Y., Huang, A. & Caro, J. Amine-modified Mg-MOF-74/CPO-27-Mg membrane with enhanced H_2/CO_2 separation. *Chem, Eng. Sci.* **124,** 27–36 (2015).

25. Li, Y. S. et al. Controllable synthesis of metal–organic frameworks: From MOF nanorods to oriented MOF membranes. *Adv. Mater.* **22,** 3322–3326 (2010).

26. Bux, H. et al. Oriented Zeolitic Imidazolate Framework-8 Membrane with Sharp H_2/C_3H_8 Molecular Sieve Separation. *J. Chem. Mater.* **23,** 2262–2269 (2011).

27. Huang, K., Dong, Z. Y., Li, Q. & Jin, W. Q. Growth of a ZIF-8 membrane on the inner-surface of a ceramic hollow fiber via cycling precursors. *Chem. Commun.* **49,** 10326–10328 (2013).

28. Knebel, A. et al. Comparative study of MIL-96 (Al) as continuous metal–organic frameworks layer and mixed-matrix membrane. *ACS Appl. Mater. Interfaces.* **8,** 7536–7544 (2016).

29. Peng, Y., Li, Y., Ban, Y. & Yang, W. Two-Dimensional Metal–Organic Framework Nanosheets for Membrane-Based Gas Separation. *Angew. Chem. Int. Edit.* **56,** 9757–9761 (2017).

30. Carta, M. et al. An efficient polymer molecular sieve for membrane gas separations. *Science* **339,** 303–307 (2013).

31. Han, S. H. et al. Tuning microcavities in thermally rearranged polymer membranes for CO_2 capture. *Phys. Chem. Chem. Phys.* **14,** 4365–4373 (2012).

32. Do, Y. S., Seong, J. G., Kim, S., Lee, J. G. & Lee, Y. M. Thermally rearranged (TR) poly (benzoxazole-co-amide) membranes for hydrogen separation derived from 3, 3′-dihydroxy-4, 4′-diamino-biphenyl (HAB), 4, 4′-oxydianiline (ODA) and isophthaloyl chloride (IPCl). *J. Membr. Sci.* **446,** 294–302 (2013).

33. Pesiri, D. R., Jorgensen, B. & Dye, R. C. Thermal optimization of polybenzimidazole meniscus membranes for the separation of hydrogen, methane, and carbon dioxide. *J. Membr. Sci.* **218,** 11–18 (2003).

34. Scholes, C. A., Smith, K. H., Kentish, S. E. & Stevens, G. W. CO_2 capture from pre-combustion processes-Strategies for membrane gas separation. *Int. J. Greenh. Gas Con.* **4,** 739–755 (2010).

35. Yang, T., Shi, G. M. & Chung, T. S. Symmetric and asymmetric zeolitic imidazolate frameworks (ZIFs)/polybenzimidazole (PBI) nanocomposite membranes for hydrogen purification at high temperatures. *Adv. Energy. Mater.* **2,** 1358–1367 (2012).

36. Yang, T., Xiao, Y. & Chung, T. S. Poly-/metal-benzimidazole nano-composite membranes for hydrogen purification. *Energy. Environ. Sci.* **4,** 4171–4180 (2011).

37. Zhu, L., Swihart, M. T. & Lin, H. Unprecedented size-sieving ability in polybenzimidazole doped with polyprotic acids for membrane H_2/CO_2 separation. *Energy Environ. Sci.* **11,** 94–100 (2018).

38. Shan, M. et al. Facile manufacture of porous organic framework membranes for precombustion CO_2 capture. *Sci. Adv.* **4,** eaau1698 (2018).

39. Kang, Z. et al. Mixed Matrix Membranes (MMMs) Comprising Exfoliated 2D Covalent Organic Frameworks (COFs) for Efficient CO_2 Separation. *Chem. Mater.* **28,** 1277–1285 (2016).

40. Fu, J. et al. Fabrication of COF-MOF Composite Membranes and Their Highly Selective Separation of H_2/CO_2. *J. Am. Chem. Soc. 138*, 7673–7680 (2016).

41. Fan, H. et al. Covalent Organic Framework–Covalent Organic Framework Bilayer Membranes for Highly Selective Gas Separation. *J. Am. Chem. Soc.* **140,** 10094–10098 (2018).

42. Ying, Y. et al. A GO-assisted method for the preparation of ultrathin covalent organic framework membranes for gas separation. *J. Mater. Chem. A* **4,** 13444–13449 (2016).

43. Tang, Y. et al. Covalent organic frameworks combined with graphene oxide to fabricate membranes for H_2/CO_2 separation. *Sep. Puri. Technol.* **223,** 10–16 (2019).

44. Das, S. & Ben, T. A [COF-300]-[UiO-66] composite membrane with remarkably high permeability and H_2/CO_2 separation selectivity. *Dalton. T* **47,** 7206–7212 (2018).

45. Wu, T., Wang, B., Lu, Z., Zhou, R. & Chen, X. Alumina-supported AlPO-18 membranes for CO2/CH4 separation. *J. Membr. Sci.* **471,** 338–346 (2014).

46. Zhou, C., Yuan, C., Zhu, Y., Caro, J. & Huang, A. Facile synthesis of zeolite FAU molecular sieve membranes on bio-adhesive polydopamine modified Al_2O_3 tubes. *J. Membr. Sci.* **494,** 174–181 (2015).

47. Huang, A., Wang, N. & Caro, J. Seeding-free synthesis of dense zeolite FAU membranes on 3-aminopropyltriethoxysilane-functionalized alumina supports. *J. Membr. Sci.* **389,** 272–279 (2012).

48. Xu, K., Yuan, C., Caro, J. & Huang, A. Silver-exchanged zeolite LTA molecular sieving membranes with enhanced hydrogen selectivity. *J. Membr. Sci.* **511,** 1–8 (2016).

49. Poshusta, J. C., Tuan, V. A., Falconer, J. L. & Noble, R. D. Synthesis and permeation properties of SAPO-34 tubular membranes. *Ind. Eng. Chem. Res.* **37,** 3924–3929 (1998).

50. Poshusta, J. C., Tuan, V. A., Pape, E. A., Noble, R. D. & Falconer, J. L. Separation of light gas mixtures using SAPO-34 membranes. *AIChE J.* **46,** 779–789 (2000).

51. Zhou, L. et al. Highly H_2 permeable SAPO-34 membranes by steam-assisted conversion seeding. *Int. J. Hydrogen Energy.* **39,** 14949–14954 (2014).

52. Li, G. et al. Thin carbon/SAPO-34 microporous composite membranes for gas separation. *J. Membr. Sci.* **374,** 83–92 (2011).

53. Kosinov, N. et al. High flux high-silica SSZ-13 membrane for CO2 separation. *J. Mater. Chem. A* **2,** 13083–13092 (2014).

54. Zhou, R. et al. Defect-Patching of Zeolite Membranes by Surface Modification Using Siloxane Polymers for CO_2 Separation. *Ind. Eng. Chem. Res.* **54,** 7516–7523 (2015).

55. Yin, X. et al. Zeolite L/carbon nanocomposite membranes on the porous alumina tubes and their gas separation properties. *J. Membr. Sci.* **348,** 181–189 (2010).

56. Yin, X., Chu, N., Yang, J., Wang, J. & Li, Z. Thin zeolite T/carbon composite membranes supported on the porous alumina tubes for CO_2 separation. *Int. J. Greenh. Gas Control.* **15,** 55–64 (2013).

57. Y. Mao. et al. Pressure-assisted synthesis of HKUST-1 thin film on polymer hollow fiber at room temperature toward gas separation. *ACS Appl. Mater. Interfaces.* **6,** 4473–4479 (2014).

58. Shah, M. N., Gonzalez, M. A., McCarthy, M. C. & Jeong, H. An unconventional rapid synthesis of high performance metal–organic framework membranes. *Langmuir* **29,** 7896–7902 (2013).

59. Hillman, F., Brito, J. & Jeong, H. Rapid one-pot microwave synthesis of mixed-linker hybrid zeolitic-imidazolate framework membranes for tunable gas separations. *ACS Appl. Mater. Interfaces*. **10,** 5586–5593 (2018).

60. He, G., Dakhchoune, M., Zhao, J., Huang, S. & Agrawal, K. V. Thin Films: Electrophoretic Nuclei Assembly for Crystallization of High-Performance Membranes on Unmodified Supports. *Adv. Funct. Mater.* **28,** 1707427 (2018).

61. Hou, J., Sutrisna, P. D., Zhang, Y. & Chen, V. Formation of ultrathin, continuous metal–organic framework membranes on flexible polymer substrates. *Angew. Chem. Int. Edit.* **55,** 3947–3951 (2016).

62. Shamsaei, E. et al. Aqueous Phase Synthesis of ZIF-8 Membrane with Controllable Location on an Asymmetrically Porous Polymer Substrate. *ACS Appl. Mater. Interfaces.* **8,** 6236–6244 (2016).

63. Ban, Y. et al. Confinement of ionic liquids in nanocages: tailoring the molecular sieving properties of ZIF-8 for membrane-based CO_2 capture. *Angew. Chem. Int. Edit.* **54,** 15483–15487 (2015).

64. Huang, A. & Caro, J. Covalent post-functionalization of zeolitic imidazolate framework ZIF-90 membrane for enhanced hydrogen selectivity. *Angew. Chem. Int. Edit.* **50,** 4979–4982 (2011).

65. Huang, A., Liu, Q., Wang, N. & Caro, J. Organosilica functionalized zeolitic imidazolate framework ZIF-90 membrane for CO_2/CH_4 separation. *Micropor. Mesopor. Mat.* **192,** 18–22 (2014).

66. Cacho-Bailo, F., Etxeberria-Benavides, M., Karvan, O., Tellez, C. & Coronas, J. Sequential amine functionalization inducing structural transition in an aldehyde-containing zeolitic imidazolate framework: application to gas separation membranes. *Crystengcomm* **19,** 1545–1554 (2017).

67. Cacho-Bailo, F., Seoane, B., Téllez, C. & Coronas, J. ZIF-8 continuous membrane on porous polysulfone for hydrogen separation. *J. Membr. Sci.* **464,** 119–126 (2014).

68. Bux, H. et al. Zeolitic imidazolate framework membrane with molecular sieving properties by microwave-assisted solvothermal synthesis. *J. Am. Chem. Soc.* **131,** 16000–16001 (2009).

69. Liu, Q., Wang, N., Caro, J. & Huang, A. Bio-inspired polydopamine: a versatile and powerful platform for covalent synthesis of molecular sieve membranes. *J. Am. Chem. Soc.* **135,** 17679–17682 (2013).

70. Pan, Y. C., Wang, B. & Lai, Z. P. Synthesis of ceramic hollow fiber supported zeolitic imidazolate framework-8 (ZIF-8) membranes with high hydrogen permeability. *J. Membr. Sci.* **421,** 292−298 (2012).

71. Zhang, X. F. et al. New membrane architecture with high performance: ZIF-8 membrane supported on vertically aligned ZnO nanorods for gas permeation and separation. *Chem. Mater.* **226,** 1975−1981 (2014).

72. Liu, Y., Wang, N., Pan, J. H., Steinbach, F. & Caro, J. In situ synthesis of MOF membranes on ZnAl-CO_3 LDH buffer layer-modified substrates. *J. Am. Chem. Soc.* **136,** 14353−14356 (2014).

73. Huang, A., Wang, N., Kong, C. & Caro, J. Organosilica-Functionalized Zeolitic Imidazolate Framework ZIF-90 Membrane with High Gas-Separation Performance. *Angew. Chem. Int. Edit.* **51,** 10551−10555 (2012).

74. Eum, K. et al. ZIF-8 Membrane Separation Performance Tuning by Vapor Phase Ligand Treatment. *Angew. Chem.Int. Edit.* **131,** 16542−16546 (2019).

75. Ghanem, B. S., Swaidan, R., Ma, X., Litwiller, E. & Pinnau, I. Energy-efficient hydrogen separation by AB-type ladder-polymer molecular sieves. *Adv. Mater.* **26,** 6696−6700 (2014).

76. Zhuang, Y. et al. Soluble, microporous, Tröger's Base copolyimides with tunable membrane performance for gas separation. *Chem. Commun.* **52,** 3817−3820 (2016).

77. Song, Q. et al. Porous organic cage thin films and molecular-sieving membranes. *Adv. Mater.* **28,** 2629−2637 (2016).

78. Biswal, B. P., Chaudhari, H. D., Banerjee, R. & Kharul, U. K. Chemically stable covalent organic framework (COF)-polybenzimidazole hybrid membranes: enhanced gas separation through pore modulation. *Chem–Eur. J.* 22, 4695−4699 (2016).

79. Lu, H. et al. A novel 3D covalent organic framework membrane grown on a porous α-Al_2O_3 substrate under solvothermal conditions. *Chem. Commun.* **51,** 15562−15565 (2015).
